# Effect of Climate Change Impact Menu Labels on Fast Food Ordering Choices Among US Adults: A Randomized Clinical Trial

**DOI:** 10.1001/jamanetworkopen.2022.48320

**Published:** 2022-12-27

**Authors:** Julia A. Wolfson, Aviva A. Musicus, Cindy W. Leung, Ashley N. Gearhardt, Jennifer Falbe

**Affiliations:** 1 Department of International Health, Johns Hopkins Bloomberg School of Public Health, Baltimore, Maryland; 2 Department of Health Policy and Management, Johns Hopkins Bloomberg School of Public Health, Baltimore, Maryland; 3 Department of Nutrition, Harvard T.H. Chan School of Public Health, Boston, Massachusetts; 4 Department of Nutritional Sciences, University of Michigan School of Public Health, Ann Arbor; 5 Department of Psychology, University of Michigan, Ann Arbor; 6 Department of Human Ecology, University of California, Davis

## Abstract

**Importance:**

There is increasing interest in strategies to encourage more environmentally sustainable food choices in US restaurants through the use of menu labels that indicate an item’s potential impact on the world’s climate. Data are lacking on the ideal design of such labels to effectively encourage sustainable choices.

**Objective:**

To test the effects of positive and negative climate impact menu labels on the environmental sustainability and healthfulness of food choices compared with a control label.

**Design, Setting, and Participants:**

This randomized clinical trial used an online national US survey conducted March 30 to April 13, 2022, among a nationally representative sample of adults (aged ≥18 years) from the AmeriSpeak panel. Data were analyzed in June to October 2022.

**Interventions:**

Participants were shown a fast food menu and prompted to select 1 item they would like to order for dinner. Participants were randomized to view menus with 1 of 3 label conditions: a quick response code label on all items (control group); green low–climate impact label on chicken, fish, or vegetarian items (positive framing); or red high–climate impact label on red meat items (negative framing).

**Main Outcomes and Measures:**

The main outcome was an indicator of selecting a sustainable item (ie, one without red meat). Secondary outcomes included participant health perceptions of the selected item and the Nutrition Profile Index (NPI) score of healthfulness.

**Results:**

Among 5049 participants (2444 female [51.6%]; 789 aged 18-29 years [20.3%], 1532 aged 30-44 years [25.9%], 1089 aged 45-59 years [23.5%], and 1639 aged ≥60 years [30.4%]; 142 Asian [5.3%], 611 Black [12.1%], and 3197 White [63.3%]; 866 Hispanic [17.2%]), high– and low–climate impact labels were effective at encouraging sustainable selections from the menu. Compared with participants in the control group, 23.5% more participants (95% CI, 13.7%-34.0%; *P* < .001) selected a sustainable menu item when menus displayed high–climate impact labels and 9.9% more participants (95% CI, 1.0%-19.8%; *P* = .03) selected a sustainable menu item when menus displayed low–climate impact labels. Across experimental conditions, participants who selected a sustainable item rated their order as healthier than those who selected an unsustainable item, according to mean perceived healthfulness score (control label: 3.4 points; 95% CI, 3.2-3.5 points vs 2.5 points; 95% CI, 2.4-2.6 points; *P* < .001; low-impact label: 3.7 points; 95% CI, 3.5-3.8 points vs 2.6 points; 95% CI, 2.5-2.7 points; *P* < .001; high-impact label: 3.5 points; 95% CI, 3.3-3.6 points vs 2.7 points; 95% CI, 2.6-2.9 points; *P* < .001). Participants in the high–climate impact label group selected healthier items according to mean (SE) NPI score (54.3 [0.2] points) compared with those in the low–climate impact (53.2 [0.2] points; *P* < .001) and control (52.9 [0.3] points; *P* < .001) label groups.

**Conclusions and Relevance:**

This randomized clinical trial’s findings suggest that climate impact menu labels, especially negatively framed labels highlighting high–climate impact items (ie, red meat), were an effective strategy to reduce red meat selections and encourage more sustainable choices.

**Trial Registration:**

ClinicalTrials.gov Identifier: NCT05482204

## Key Points

**Question:**

What effects do positive and negative climate impact menu labels have on the environmental sustainability of adult restaurant food choices in a nationally representative sample?

**Findings:**

In this randomized clinical trial with 5049 US adults, 23% more participants in the high–climate impact label condition ordered a sustainable (ie, non–red meat) item and 10% more participants in the low–climate impact label condition ordered a sustainable item compared with the control group.

**Meaning:**

These findings suggest that climate impact menu labels may be an effective strategy to promote more sustainable restaurant food choices and that labels highlighting high–climate impact items may be most effective.

## Teaser

This randomized clinical trial investigates the effect of positive and negative climate impact menu labels on the environmental sustainability of fast food ordering choices among US adults.

## Introduction

Animal-based food production, primarily driven by beef production, is responsible for 14.5% of global greenhouse gas emissions (GHGEs) and is an important modifiable contributor to climate change.^1,2,3,4^ In the United States, meat consumption, red meat consumption in particular, consistently exceeds recommended levels based on national dietary guidelines.^1,5,6^ Shifting current dietary patterns toward more sustainable diets with lower amounts of red meat consumed could reduce diet-related GHGEs by up to 55%.^7^ More environmentally sustainable diets containing less red meat could also have health benefits; evidence links consumption of red meat with increased risk of mortality, stroke, colon cancer, and type 2 diabetes.^8,9,10^

Fast food restaurants are an important source of red meat in the US diet; on a typical day, more than one-third of US individuals consume fast food, which is associated with numerous adverse health outcomes.^11,12^ Therefore, fast food restaurants are an important setting to encourage more environmentally sustainable dietary choices.^13^ One strategy to encourage such choices is the use of sustainability labels on restaurant menus indicating the climate impact of each menu item based on its associated GHGEs, or carbon footprint. Restaurants and cafeterias across the US are increasingly implementing sustainability labels,^14,15^ but label designs are not consistent across settings, and there is a lack of data on how these labels should be best designed to help consumers make more informed choices. Previous research^16,17,18,19,20,21,22^ suggests that labels providing a negative signal that an item is environmentally unsustainable (ie, has high GHGEs) may be more effective than those providing a positive signal that an item is environmentally sustainable (ie, has low GHGEs). However, this has not been tested in a fast food restaurant setting to our knowledge.

Aside from sustainability labels’ impact on the environmental sustainability of food choices, it is important to consider their effect on the healthfulness of food choices. Evidence suggests that products labeled as environmentally sustainable may be perceived as healthier,^23^ which is problematic in the restaurant setting, where many of the more sustainable (ie, non–red meat-based) items are still high in calories, saturated fat, added sugar, and salt.^24^ Thus, positively framed sustainability labels on unhealthy items could mislead consumers to perceive unhealthy foods as healthy, thereby encouraging consumption of these items. However, the extent that positively or negatively framed sustainability labels influence perceptions of healthfulness of fast food menu items is currently unknown.

The objective of this study was to test the effect of positive and negative sustainability labels compared with quick response (QR) code (ie, control) labels on the environmental sustainability of adults’ fast food restaurant menu choices. Secondary objectives were to examine labels’ effects on the healthfulness of menu choices and health perceptions of menu items. We hypothesized that participants would be more likely to select sustainable options when viewing menus with positive or negative framing compared with control labels but that in the positive condition, menu selections would not be healthier compared with other conditions. We also hypothesized that participants exposed to positively framed labels would perceive their selected items to be healthier compared with individuals in other conditions.

## Methods

**Figure 1.**
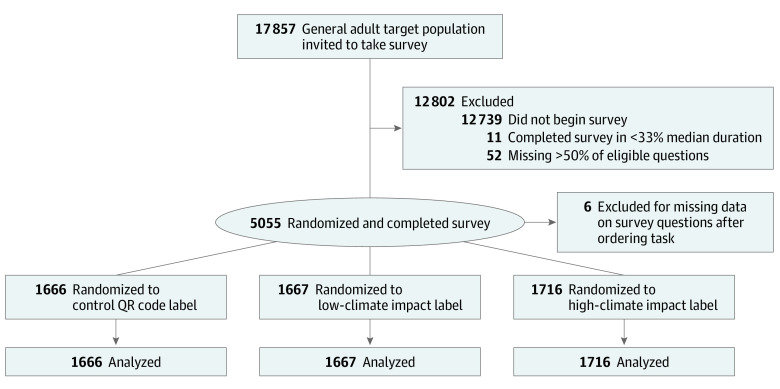
Study Flow Chart QR indicates quick response.

This randomized clinical trial was approved by the Johns Hopkins Bloomberg School of Public Health Institutional Review Board (IRB) and registered on ClinicalTrials.gov (NCT05482204). Participants provided informed consent. The study followed the Consolidated Standards of Reporting Trials (CONSORT) reporting guideline for randomized clinical trials (Figure 1). Trial protocol and analysis plan as submitted to the IRB are available in Supplement 1.

### Participants

Participants were recruited for an online randomized clinical trial from the National Opinion Research Center (NORC) AmeriSpeak panel.^25^ In this probability-based, nationally representative online panel, participating households are randomly selected with a known, nonzero probability from the NORC National Frame, as well as via address-based sample frames covering approximately 97% of the US household population. Participants are recruited by mail, telephone, and field interviewers.^25^ The panel currently includes more than 54 000 individuals in more than 43 000 households.^25^ Participants typically respond to 1 to 2 surveys per month and were compensated the equivalent of $2 for this survey. The survey-embedded experiment for this study was conducted from March 30 to April 13, 2022, among adults (aged ≥18 years) in the US. The a priori target number for the completed survey was 5000 participants, which authors calculated would provide more than 80% power to detect an effect size of 0.12 for the primary outcome. Participants were excluded for poor data quality if they completed the survey in less than one-third the median completion time of 1 minute (11 individuals) or failed to answer more than 50% of questions (52 individuals). Of 17 857 panelists invited to participate, 5055 individuals completed the survey, for a survey completion rate of 28.3%. We excluded 6 participants due to missing data on survey questions after the ordering task, for a final analytic sample of 5049 adults.

### Survey Procedures and Randomization

**Figure 2.**
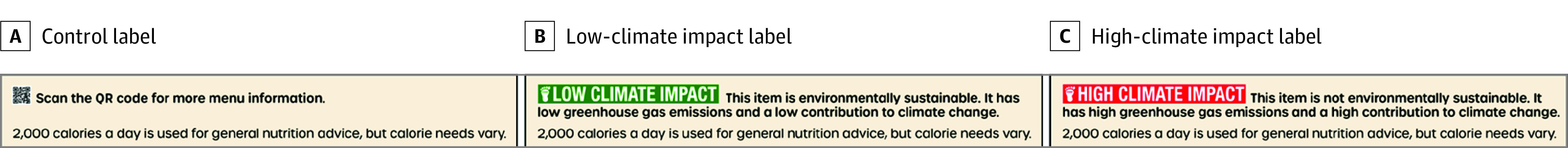
Menu Labels by Experimental Condition

After providing informed consent, participants were instructed to imagine they were in a restaurant and about to order dinner. They were then shown a fast food menu and instructed to select 1 item they wanted to order for themselves. The menu was designed to mimic a large fast food restaurant chain in the US and displayed 14 entrée items, including beef and plant-based meat substitute (Impossible Foods) hamburgers, chicken and fish sandwiches, chicken nuggets, and salads. For the menu ordering task, participants were randomized 1:1:1 to view 1 of 3 menus, each with a different label: (1) QR code labels on all items (control); (2) green low–climate impact labels on chicken, fish, or vegetarian items (positive framing); or (3) red high–climate impact labels on beef items (negative framing). Menu prices were based on real-world prices taken from the model restaurant menu at the time the menu was designed. Prices were constant across menu conditions. The top of each menu featured a disclosure statement explaining the label’s meaning. The low–climate impact condition menu stated, “This item is environmentally sustainable. It has low greenhouse gas emissions and a low contribution to climate change.” The high–climate impact condition menu stated, “This item is not environmentally sustainable. It has high greenhouse gas emissions and a high contribution to climate change” (Figure 2). After selecting an item, participants answered a brief questionnaire.

### Measures

#### Primary Outcome

The primary outcome was an indicator of whether the menu item selected by the participant was sustainable (ie, did not contain red meat, labeled as low climate impact in condition 2). The outcome regarding healthfulness of items that participants ordered was changed to a secondary outcome because no menu items met criteria to be considered healthy (Nutrition Profile Index [NPI] score >64). We also assessed the proportion of participants who selected each type of food item (beef hamburger, plant-based hamburger, chicken or fish item, or salad) to further understand label effects on ordering choices.

#### Nutrition Profile Index Score of Selected Menu Item

The NPI is a validated nutrition quality measure based on the UK Ofcom nutrient profiling model, which was used previously to quantify the nutrition quality of fast food menu items.^26,27,28,29^ The NPI provides a single score based on positive and negative nutrients per 100 g of an individual food item.^30^ Details about NPI scoring are available elsewhere.^30,31^ Briefly, points are awarded for positive nutrients (ie, fruit, vegetables, and nuts; fiber; and protein) and subtracted for negative nutrients (ie, energy, saturated fat, total sugar, and sodium). This score is converted to a 0 (least healthy) to 100 (healthiest) NPI score using the following formula: NPI score = [−2] × OfcomNP score + 70. Items with scores of 64 or greater are considered healthy.^31^ To calculate the NPI, we used nutrition and serving size information from a fast food chain restaurant website (Burger King) accessed in June 2022.^32^ For the 2 items not listed on the website, we used nutrition information from Fast Food Nutrition, an independent website that publishes nutrition facts for 90 of the largest fast food chains in the US.^33^

#### Health Perceptions of Selected Item

After the ordering task, participants were asked, “How unhealthy or healthy do you think the meal you ordered is?” They could respond on a 7-point scale, from 1 = “very unhealthy” to 7 = “very healthy.”

#### Noticing Labels

After the ordering task, participants were asked, “Did you notice any labels (other than calories) next to or below the menu items?” Response options were no; yes, a sugar label; yes, a sodium label; yes, a QR code label; yes, a climate impact label; yes, a grown in the US label. We used 2 binary indicators to measure whether the participant affirmed that they noticed any label and whether the participant noticed the correct label for their assigned condition.

#### Perceived Message Effectiveness

A single item was adapted from the validated 3-item University of North Carolina Perceived Message Effectiveness (PME) scale, which measures the potential of a message to change behavior.^34^ We adapted the discouragement item to ask participants how much they agreed or disagreed (on a 5-point scale from strongly disagree to strongly agree) that “Information on the menu discouraged me from wanting to consume menu items with a high impact on climate change.” The PME measure has been adapted and used in numerous prior studies of menu and food labeling experiments and has been shown to be associated with behavior.^35,36,37,38,39^

#### Attitudes About Climate Change and Healthy Eating

At the end of the survey, participants reported how much they agreed or disagreed (on a 5-point scale) with 2 adapted statements. These statements were “I am concerned about climate change” and “Making healthy food and beverage choices is important to me.”^40^

#### Sociodemographic Characteristics

Participants reported sociodemographic characteristics upon enrollment in the panel. Characteristics included sex, age, race and ethnicity, education, annual household income, region of residence, urbanicity, and political ideology. All study characteristics, including race and ethnicity, were included to examine potential heterogeneous effects of the labels by participant characteristic or sociodemographic factor. Options for ethnicity were Hispanic and non-Hispanic, and options for race were non-Hispanic American Indian or Alaska Native, non-Hispanic Asian, non-Hispanic Black, non-Hispanic White, and non-Hispanic identities specified in open-text responses. Race and Hispanic identity were asked separately. Participants could select as many race and ethnicity categories as applied to them in response to the race and ethnicity identity questions. Those who checked multiple categories were coded as non-Hispanic multiracial if they did not identify as Hispanic. Due to small numbers, non-Hispanic American Indian or Alaska Native, non-Hispanic identities specified in open-text responses that did not fit in an existing racial category, and missing race were combined as non-Hispanic other race.

### Statistical Analysis

Study-specific survey weights provided by AmeriSpeak were used to generate nationally representative estimates for analyses. Logistic regression was used to examine the probability of selecting a sustainable menu item by experimental condition. To explore potential heterogeneous effects by sociodemographic characteristic, interactions between experimental condition and sex, age, race and ethnicity, household income, political party, and attitudes about climate change and healthy eating were explored. To examine potential health halo effects, linear regression with an interaction between experimental condition and whether a sustainable item was selected was used to estimate differences in perceptions of healthfulness of the selected meal.

In supplemental analyses, logistic regression was used to estimate the probability of seeing any label and seeing the correct label by experimental condition. Linear regression was used to estimate PME by experimental condition. Multinomial logistic regression was used to estimate the probability of selecting a beef hamburger, plant-based hamburger, chicken or fish item, or salad by experimental condition. Finally, linear regression was used to estimate the actual healthfulness (by NPI score) of the selected meal and PME by experimental condition.

Postestimation margin commands were used to report predicted probabilities or predicted means for analyses. We conducted analyses using Stata statistical software version 17 (StataCorp) in June to October 2022. Tests were 2 sided and significance was considered at *P* < .05.

## Results

There were 5049 participants (2444 female [51.5%; 95% CI, 49.7%-53.3%]; 789 aged 18-29 years [20.3%; 95% CI, 18.7%-22.0%], 1532 aged 30-44 years [25.9%; 95% CI, 24.4%-27.4%], 1089 aged 45-59 years [23.5%; 95% CI, 21.9%-25.1%], and 1639 aged ≥60 years [30.4%; 95% CI, 28.8%-32.0%]; 142 non-Hispanic Asian [5.3%; 95% CI, 4.3%-6.6%], 611 non-Hispanic Black [12.1%; 95% CI, 10.9%-13.2%], and 3197 non-Hispanic White [63.3%; 95% CI, 60.7%-64.3%]; 866 Hispanic [17.2%; 95% CI, 15.5%-18.4%]) (Table). Demographic percentages are weighted. Individuals in low–impact (49.4%; 95% CI: 46.3%-52.5%) and high–impact (48.7%; 95% CI, 45.5%-51.9%; *P* < .001) climate-label conditions were more likely than those in the control condition (21.5%; 95% CI, 18.9%-24.1%; *P* < .001) to report noticing any label; they were also more likely to report seeing their assigned label (low-impact label: 43.9%; 95% CI, 40.8%-47.0%; high-impact label: 42.8%; 95% CI, 39.7%-46.0%; control label: 17.1%; 95% CI, 14.7%-19.5%; all *P* < .001). Among participants who reported noticing a label on the menu, nearly 90% in both label conditions correctly reported seeing their assigned label (low-impact label: 89.0%; 95% CI, 85.9%-92.0%; high-impact label: 88.0%; 95% CI, 85.0%-91.0%) (eFigure 1 in Supplement 2). Individuals in climate impact label conditions were more likely than those in the control condition (mean score, 2.1; 95% CI, 2.0-2.2) to report that the label discouraged consumption of high–climate impact label items, with perceived discouragement higher in the high–climate impact label (mean score, 2.7; 95% CI, 2.6-2.8) than low–climate impact label condition (mean score, 2.4; 95% CI, 2.3-2.5) (all *P* < .001) (eFigure 2 in Supplement 2).

**Table.** Participant Characteristics

Characteristic	Participants, No. (weighted % [95% CI])			
	Overall (N = 5049)	Label condition		
		Control (n = 1666 [32.0%])	Low impact (n = 1667 [33.2%])	High impact (n = 1716 [34.9%])
Sex				
Male	2605 (48.5 [46.7-50.3])	856 (48.1 [44.9-51.2])	857 (48.0 [44.9-51.1])	892 (49.4 [46.2-52.6])
Female	2444 (51.5 [49.7-53.3])	810 (52.0 [48.8-55.1])	810 (52.0 [48.9-55.1])	824 (51.5 [47.4-53.8])
Age, y				
18-29	789 (20.3 [18.7-22.0])	270 (21.0 [18.2-24.0])	261 (19.1 [16.6-21.9])	258 (20.8 [17.9-24.0])
30-44	1532 (25.9 [24.4-27.4])	496 (25.0 [22.4-27.7])	527 (28.7 [26.0-31.5])	509 (24.1 [21.7-26.7])
45-59	1089 (23.5 [21.9-25.1])	364 (23.5 [20.9-26.4])	354 (22.8 [20.3-25.6])	371 (24.0 [21.4-26.8])
≥60	1639 (30.4 [28.8-32.0])	536 (30.5 [27.8-33.4])	525 (29.4 [26.7-32.3])	578 (31.1 [28.4-34.1])
Race and ethnicity				
Hispanic	866 (16.9 [15.5-18.4])	280 (16.1 [13.8-18.6])	290 (16.5 [14.2-19.1])	296 (18.0 [15.6-20.6])
Non-Hispanic				
Asian	142 (5.3 [4.3-6.6])	44 (4.6 [3.2-6.7])	48 (4.8 [3.5-6.7])	50 (6.5 [4.5-9.2])
Black	611 (12.0 [10.9-13.2])	221 (14.2 [12.1-16.6])	196 (11.4 [9.6-13.4])	194 (10.6 [8.8-12.6])
White	3197 (62.5 [60.7-64.3]	1051 (62.7 [59.4-65.8])	1,046 (63.0 [59.9-66.0])	1110 (61.9 [58.6-65.1])
Other^a^	70 (1.0 [0.7-1.4])	24 (0.8 [0.4-1.3])	26 (1.4 [0.8-2.3])	20 (1.9 [0.5-1.6])
Multiracial^b^	163 (2.3 [1.8-2.8])	46 (1.7 [1.1-2.6])	61 (2.8 [2.0-4.0])	56 (2.2 [1.5-3.2])
Education				
<High school	242 (9.6 [8.3-11.1])	69 (8.6 [6.6-11.3])	87 (10.3 [8.2-12.9])	86 (9.8 [7.6-12.7])
High school graduate or equivalent	846 (28.3 [26.5-30.2])	269 (27.2 [24.1-30.5])	279 (27.6 [24.6-30.8])	298 (30.1 [27.0-33.5])
Vocational school	2224 (27.0 [25.7-28.4])	755 (28.2 [25.8-30.7])	714 (26.2 [24.0-28.6])	755 (26.7 [24.4-29.1])
Bachelor’s degree	1014 (19.9 [18.5-21.3])	326 (19.8) 17.5-22.3	361 (21.8 [19.4-24.3])	327 (18.2 [16.0-20.6])
Postgraduate or professional degree	723 (15.2 [14.0-16.5])	247 (16.2 [14.1-18.7])	226 (14.2 [12.2-16.3])	250 (15.2 [13.2-17.4])
Household annual income, $				
<30 000	1193 (25.3 [23.6-26.9])	418 (27.4 [24.6-30.5])	381 (23.9 [21.2-26.8])	394 (24.5 [21.8-27.5])
30 000 to <60 000	1326 (24.4 [22.8-26.0])	436 (23.9 [21.3-26.8])	446 (25.2 [22.6-27.9])	444 (24.0 [21.4-26.8])
60 000 to <100 000	1344 (25.8 [24.3-27.4])	443 (25.1 [22.5-27.8])	423 (25.2 [22.5-28.1])	478 (27.1 [24.4-29.9])
≥100 000	1186 (24.6 [23.0-26.2])	369 (23.6 [21.0-26.4])	417 (25.8 [23.2-28.5])	400 (24.4 [21.7-27.3])
Region				
Northeast	699 (17.2 [15.8-18.7])	243 (18.4 [15.8-21.3])	223 (16.1 [13.8-18.6])	233 (17.2 [14.8-19.9])
Midwest	1332 (20.6 [19.3-22.0])	467 (21.9 [19.7-24.4])	417 (20.5 [18.2-22.9])	448 (19.6 [17.5-21.9])
South	1723 (38.3 [36.5-40.1])	541 (36.5 [33.4-39.7])	589 (39.1 [36.1-42.3])	593 (39.1 [35.9-42.3])
West	1295 (23.9 [22.4-25.5])	415 (23.2 [20.7-25.9])	438 (24.3 [21.8-27.1])	442 (24.2 [21.5-27.1])
Urbanicity				
Nonmetro	744 (16.2 [14.9-17.6])	245 (15.9 [13.8-18.4])	246 (16.3 [14.1-18.8])	253 (16.3 [14.0-18.8])
Metro	4305 (83.8 [82.4-85.1])	1421 (84.1 [81.7-86.2])	1421 (83.7 [81.2-85.9])	1463 (83.7 [81.2-86.0])
Political ideology				
Very liberal	677 (12.2 [11.1-13.4])	233 (12.2 [10.4-14.4])	202 (11.3 [9.6-13.4])	242 (13.0 [11.0-15.2])
Somewhat liberal	691 (13.9 [12.6-15.3])	242 (15.2 [13.0-17.7])	227 (13.2 [11.3-15.5])	222 (13.4 [11.2-15.9])
Moderate	2,064 (41.7 [39.9-43.5])	663 (41.2 [38.0-44.4])	683 (41.7 [38.6-44.9])	718 (42.1 [38.9-45.3])
Somewhat conservative	924 (19.0 [17.6-20.6])	293 (19.2 [16.7-22.0])	325 (19.2 [16.9-21.8])	306 (18.7 [16.3-21.4])
Very conservative	634 (13.2 [12.0-14.5])	216 (12.2 [10.4-14.3])	217 (14.5 [12.4-16.9])	201 (12.9 [10.9-15.2])
I am concerned about climate change				
Strongly disagree	572 (11.0 [9.9-12.2])	202 (12.1 [10.1-14.5])	192 (11.9 [10.0-14.2])	178 (9.2 [9.6-11.0])
Somewhat disagree	463 (8.9 [8.0-10.0])	153 (9.4 [7.8-11.4])	170 (9.1 [7.5-11.0])	140 (8.3 [6.7-10.3])
Neither agree nor disagree	890 (18.8 [17.4-20.4])	299 (19.7 [17.1-22.5])	293 (18.4 [16.1-21.0])	298 (18.5 [16.1-21.1])
Somewhat agree	1607 (32.1 [30.4-33.9])	523 (31.2 [28.4-34.3])	539 (32.4 [29.6-35.5])	545 (32.6 [29.6-35.8])
Strongly agree	1517 (29.1 [27.5-30.7])	489 (27.5 [24.9-30.3])	473 (28.1 [25.5-31.0])	555 (31.4 [28.5-34.4])
Making healthy food and beverage choices is important to me				
Strongly disagree	120 (2.6 [2.0-3.3])	36 (2.7 [1.7-4.1])	45 (2.6 [1.8-3.8])	39 (2.5 [1.5-4.0])
Somewhat disagree	271 (5.8 [5.0-6.8])	102 (7.6 [5.9-9.6])	77 (5.1 [3.9-6.8])	92 (4.8 [3.7-6.2])
Neither agree nor disagree	912 (18.7 [17.3-20.2])	303 (18.3 [15.9-20.9])	303 (18.8 [16.4-21.3])	306 (19.1 [16.6-21.7])
Somewhat agree	2,372 (45.8 [44.0-47.6])	805 (46.6 [43.4-49.8])	787 (47.0 [43.9-50.1])	780 (43.9 [40.8-47.1])
Strongly agree	1,374 (27.1 [25.5-28.8])	420 (24.9 [22.3-27.7])	455 (26.5 [23.9-29.3])	499 (29.7 [26.8-32.8])

^a^ The Non-Hispanic other race category included the following due to small numbers: non-Hispanic American Indian or Alaska Native, non-Hispanic identities specified in open-text responses that did not fit in an existing racial category, or missing race.

^b^ Participants could check all categories that applied in response to the racial identity question. Those who checked multiple categories were coded as non-Hispanic multiracial if they did not identify as Hispanic, which was asked separately.

**Figure 3.**
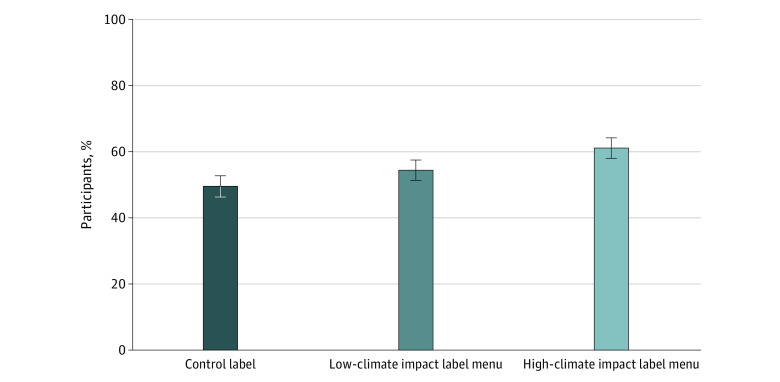
Predicted Probability of Selecting a Sustainable Menu Item by Experimental Condition Results are among 5049 participants and are based on postestimation margins after weighted simple logistic regression. Whiskers indicate 95% CIs.

Climate impact labels were effective at encouraging sustainable selections from the menu (Figure 3). In the high–impact and low–impact-label conditions, respectively, 61.1% (95% CI, 58.0%-64.2%; *P* < .001) and 54.4% (95% CI, 52.3%-57.5%; *P* = .03) of participants ordered a sustainable item compared with 49.5% (95% CI, 46.3%-52.7%) of participants in the control condition. Thus, compared with participants in the control group, 23.5% more participants (95% CI, 13.7%-34.0%; *P* < .001) selected a sustainable menu item when menus displayed high–climate impact labels and 9.9% more participants (95% CI, 1.0%-19.8%; *P* = .03) selected a sustainable menu item when menus displayed low–climate impact labels. Compared with participants in the low–climate impact label condition, more participants in the high–climate impact label condition made a sustainable choice (*P* = .003). The only significant interaction found was between condition and sex (ie, male vs female participants; high-impact label: 53.7%; 95% CI, 49.1%-58.3% vs 68.4%; 95% CI, 64.2%-72.5%; low-impact label: 53.4%; 95% CI, 50.0%-57.8% vs 55.3%; 95% CI, 50.9%-59.7%; control label: 47.5%; 95% CI, 43.0%-52.1% vs 51.4%; 95% CI, 46.9%-55.8%; *P* for interaction = .02) (eFigure 3 in Supplement 2), with women more responsive to the labels, particularly the high-impact label.

The predicted probability of selecting a beef hamburger, plant-based hamburger, chicken or fish item, or salad by experimental condition is shown in eFigure 4 in Supplement 2. Compared with participants in the control group (11.1%; 95% CI, 9.1%-13.0%), participants in climate–impact-label conditions were more likely to order salad items (low-impact label: 15.2%; 95% CI, 12.9%-17.5%; *P* = .007; high-impact label: 15.9%; 95% CI, 13.4%-18.4%; *P* = .003).

**Figure 4.**
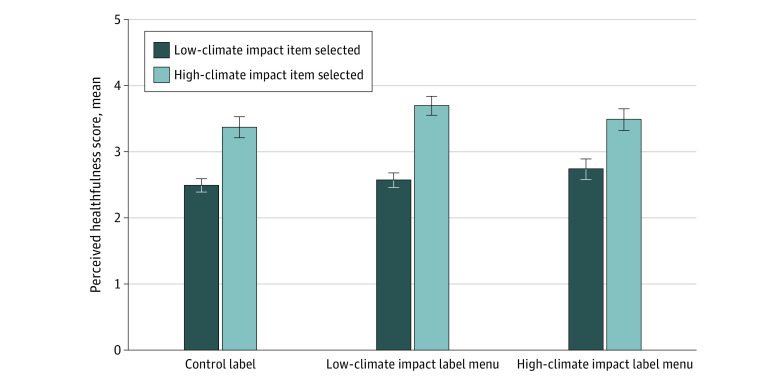
Perceptions of Healthfulness of the Item Selected Results are based on postestimation margins after linear regression with interaction between experimental condition and an indicator for selecting a sustainable (ie, lower–climate impact) menu item. Perceived healthfulness score is a 7-point scale, from 1 = very unhealthy to 7 = very healthy. Whiskers indicate 95% CIs.

No menu items met the threshold to be considered healthy, and NPI scores ranged from 36 points, for a beef hamburger, to 62 points for a plant-based hamburger (eTable in Supplement 2). Items selected by individuals in the high–climate impact label condition had higher mean (SE) NPI scores (reflecting healthier choices) compared with items selected by those in the low–climate impact label condition (54.3 [0.2] points vs 53.2 [0.2] points; *P* < .001) and control label condition (52.9 [0.3] points; *P* < .001) (eFigure 5 in Supplement 2). Among individuals who selected a sustainable item, those in the low–climate impact label condition perceived their item to be significantly healthier than participants in the control condition (Figure 4). Additionally, mean scores for perceived healthfulness indicate that participants correctly perceived that the menu item they selected was not healthy. However, perceptions of healthfulness of the selected item were higher for individuals who selected a sustainable item compared with those who did not regardless of label condition, according to mean perceived healthfulness score (control label: 3.4 points; 95% CI, 3.2-3.5 points vs 2.5 points; 95% CI, 2.4-2.6 points; *P* < .001; low-impact label: 3.7 points; 95% CI, 3.5-3.8 points vs 2.6 points; 95% CI, 2.5-2.7 points; *P* < .001; high-impact label: 3.5 points; 95% CI, 3.3-3.6 points vs 2.7 points; 95% CI, 2.6-2.9 points; *P* < .001). Among individuals who selected a high–climate impact (ie, beef) item, participants in the high–climate impact label condition perceived their selection to be healthier compared with those in the control label condition (*P* = .01). Among individuals who selected a low–climate impact item, participants in the low–climate impact label condition perceived their selection to be healthier compared with those in the control label condition (*P* = .003).

## Discussion

In this randomized clinical trial conducted online with a large nationally representative sample, we found that climate impact menu labels were effective compared with a QR code label at encouraging US adults to choose a more environmentally sustainable (ie, non–red meat) item from a fast food restaurant menu. Furthermore, we found that labeling red meat items with negatively framed, red high–climate impact labels was more effective at increasing sustainable selections than labeling non–red meat items with positively framed, green low–climate impact labels.

While individuals who viewed the menu with high–climate impact labels selected healthier items compared individuals in the control group, which was consistent with our hypothesis, this was not the case for individuals in the low–climate impact condition. However, individuals in the low–climate impact condition, particularly those who selected sustainable items, perceived that their selection was healthier than those in the control condition. This health halo effect may be important because many sustainable items are not particularly healthy (no menu items in this study met the threshold to be considered healthy based on NPI scores), and the health halo effect may encourage their overconsumption.^41^ Labels signaling the climate or environmental impact of food products have emerged as a potential strategy to promote sustainable food choices in restaurant, cafeteria, and supermarket settings.^23,39,42,43,44^ Consistent with prior message framing research,^16,19,20,21^ findings from this study suggest that sustainability labels, particularly labels warning of high climate impact on red meat items in fast food restaurants, may be an effective means of promoting more environmentally sustainable choices. However, the impact of climate labels on the healthfulness of food intake may be mixed depending on framing. High–climate impact labels may be more effective for additionally encouraging sustainable and potentially healthier choices, while low–climate impact labels may lead to misperceptions about the healthiness of food choices. As sustainability becomes increasingly marketed to consumers in restaurant settings, an undeserved health halo conferred to unhealthy menu items may encourage their overconsumption, which could have negative implications for diet quality, obesity, and other diet-related health outcomes.^11,41,45^

Although meat consumption in the US remains high,^46^ the restaurant industry has documented increasing consumer demand for vegan, vegetarian, and plant-based items.^47^ Several major fast food chain restaurants recently introduced meat-free or meat-alternative menu items,^48,49^ and restaurant industry reports identified increasing sustainability as among the top restaurant menu trends in 2020.^50^ Importantly, voluntary industry sustainability labels implemented to date have been positive labels indicating low-GHGE or sustainable items (eg, the Cool Food pledge recently adopted by Panera),^14^ rather than traffic light systems (in which food items are labeled with a traffic light symbol, with green indicating a healthy item, yellow indicating an item with marginal healthfulness or to be consumed in moderation, and red indicating an unhealthy item) or negative, high–climate impact warning labels. However, our results, consistent with those of other warning label studies,^20,21,22^ suggest that a negatively framed, high–climate impact label may be more effective. It is unlikely that industry would voluntarily adopt a negatively framed label approach; such an approach may need to be mandated or incentivized via legislation or regulation. However, negatively framed, high–climate impact labels may easily be adopted in settings like workplaces, universities, hospitals, and other anchor institutions with carbon neutrality commitments.

It is notable that we did not observe differences in label effects by sociodemographic characteristics other than sex, for which the effect of high–climate impact labels was stronger for female participants. It is especially surprising that the effects of climate impact labels did not differ based on underlying concerns about climate change or health; however, this is consistent with results from another online experimental study.^39^ Despite the large sample size in our study, we may not have had sufficient power to detect subgroup differences. This is an important area for future research.

### Limitations

This study has several limitations. The online experiment measured one-time, hypothetical selections rather than actual food purchases and consumption from an in-person fast food restaurant setting. Participants may also have been susceptible to social desirability bias, so results may overstate label effects on shifting food choices; however, this was minimized by anonymity. In addition, participants were exposed to the labels only once; in real-world settings with repeated exposures to climate impact labels, effects may be greater. Relatedly, the online menu used had a limited selection of items and did not include side dishes or beverages; future research should explore label effects on overall healthfulness and sustainability of full meals ordered across a variety of restaurants that offer varying levels of red and non–red meat menu items and overall meal costs. Additionally, only 1 item from the PME measure was used, which limits understanding of all dimensions of perceived message effects. Future research should test more label designs using qualitative and quantitative research on how people understand different climate impact labels and messages, including traffic light labeling schemes, and evaluate them in online and in-person fast food and other settings.

## Conclusions

In response to dual concerns of climate change and human health, a growing body of research is focused on strategies to reduce consumption of red meat and encourage more sustainable options. Restaurants are increasingly introducing new sustainable menu items and are marketing sustainability. However, sustainability-focused menu labels have, to date, been implemented in few settings. Results from this randomized clinical trial suggest that fast food menu sustainability labels (with positive and negative framing) encouraged more sustainable food choices compared with control labels, and high–climate impact labels on menu items that had high GHGEs were the most effective. Results also suggest that sustainability labels, particularly positively framed, low–climate impact labels, may confer an undeserved health halo effect on unhealthy food items. Future research should investigate different label designs to promote sustainable food choices and evaluate effects on sustainability and healthfulness of food choices in fast food restaurants and other settings.

## Notes

Supplement 1.
Trial Protocol

Supplement 2. **eFigure 1.** Proportion of Respondents Who Reported Noticing Labels on Menu by Treatment Condition **eFigure 2.** Perceptions That Label Discouraged Consumption of Menu Items With High Impact on Climate Change **eFigure 3.** Predicted Probability of Selecting Sustainable Menu Item by Treatment Condition and Sex **eFigure 4.** Predicted Probability of Selecting Beef Hamburger, Plant-Based Hamburger, Chicken or Fish Item, or Salad by Experimental Condition **eTable.** Nutrition Profile Index Score and Calorie Content of Menu Items **eFigure 5.** Predicted Mean Nutrition Profile Index Score of Ordered Item by Experimental Condition

Supplement 3.
Data Sharing Statement
