# Error in Figure 4

**DOI:** 10.1001/jamanetworkopen.2022.55433

**Published:** 2023-01-17

In the Original Investigation titled “Effect of Climate Change Impact Menu Labels on Fast Food Ordering Choices Among US Adults: A Randomized Clinical Trial,”^1^ published December 27, 2022, there was an error in Figure 4. The legend mislabeled what each color represented. This article has been corrected.^1^

## References

[1] Wolfson JA, Musicus AA, Leung CW, Gearhardt AN, Falbe J. Effect of climate change impact menu labels on fast food ordering choices among US adults: a randomized clinical trial. JAMA Netw Open. 2022;5(12):e2248320. doi:10.1001/jamanetworkopen.2022.48320 36574248 PMC9857560

