# Supplementary material for: Effect of Climate Change Impact Menu Labels on Fast Food Ordering Choices Among US Adults: A Randomized Clinical Trial
Source: JAMA Netw Open. 2022 Dec 27;5(12):e2248320. doi: 10.1001/jamanetworkopen.2022.48320 (PMC9857560; doi:10.1001/jamanetworkopen.2022.48320)
Supplement: Supplement 1. — Trial Protocol [file jamanetwopen-e2248320-s001.pdf]

JHSPH IRB Research Plan for New Data Collection
21Sep2021

For new data collection, new data collection plus secondary data analysis, biospecimen repositories, and data coordinating center protocols.

PI Name: Julia Wolfson
Study Title: Do sustainability labels lead to more sustainable and healthier food choices'
IRB No.: IRB00018722
PI Version No. / Date: V3/ November 29, 2021

I. Aims of the Study: *Describe the aims/objectives of the research and/or the project's research questions or hypotheses.*

We have two primary research questions: 1) Do positive sustainability labels on low-GHGE fast-food menu items encourage more sustainable ordering choices compared to negative labels on high-GHGE items or QR code control labels? 2) Do positive sustainability labels on low-GHGE fast-food menu items encourage healthier choices compared to negative labels on high-GHGE items or QR code control labels? We hypothesize that: 1) participants will be more likely to order sustainable options when they are positively labeled as sustainable, but 2) they will not necessarily be healthier compared to the other conditions.

II. Background and Rationale: *Explain why this study is being done. Summarize briefly what is already known about the issue and reference previously published research, if relevant.*

Climate change and diet-related non-communicable diseases (including many cancers, obesity, diabetes, and heart disease) are among the most pressing global public health issues. The modern food system is responsible for more than 30% of global anthropogenic Greenhouse Gas Emissions (GHGE), including 14.5% of global GHGE from food animal production alone. In the United States (U.S), meat consumption, and red meat consumption in particular, is consistently far above recommended levels based on national Dietary Guidelines. On a typical day, more than one-third of Americans eat out in fast-food restaurants which are a common source of red meat in American's diets and are associated with numerous adverse health outcomes. Therefore, fast-food restaurants will be a key environment for any efforts to shift Americans towards a 'win-win' 'planetary-health' diet that benefits both climate change and individual health. Similar to calorie menu labels, which have been a key public health tool to facilitate healthier choices in restaurant settings, menu labels that either provide a negative signal that an item is environmentally unsustainable (i.e. high GHGE), or menu labels that provide a positive signal that an item is an environmentally sustainable choice (i.e., low GHGE) are a potential tool to shift consumer food choices towards a 'planetary-health' diet. evidence suggests that products labeled as environmentally sustainable receive a 'health halo' even in unrelated domains, like improved perceptions of health and tastiness. Whether sustainability-focused menu labels (either negative or positive) are effective at encouraging sustainable food choices in fast-food restaurants, and whether such labels also encourage healthier food choices, has never been tested. Therefore, in this study we will test whether sustainability labels on a fast-food restaurant menu nudge consumers towards more environmentally sustainable and/or healthier food choices by comparing two labels, a red negative nudge label [HIGH CLIMATE IMPACT] displayed on high-GHGE items and a green positive nudge label [LOW CLIMATE IMPACT] on low-GHGE items. We will also compare effects of these labels against a control condition with a Quick Response (QR) code label on all menu items.

III. Study Design:

A. *Provide a BRIEF overview of your study design and methods. The study design must relate to your stated aims/objectives. DETAILS WILL BE REQUESTED LATER. If your study also involves analysis*

of existing data, please complete Section XI, "Secondary Data Analysis of Existing Data" in the last part of this research plan. If your study ONLY involves analysis of existing data, please use the research plan template for secondary data analysis (JHSPH IRB Research Plan for Secondary Data Analysis of Existing Data/Specimens).

We will conduct a randomized controlled experiment in which participants are randomly assigned to view one of three versions of a fast-food menu based on a well-known burger-focused fast-food chain, and instructed to select an entrée they would hypothetically like to order for dinner. We will also ask questions about the perceived message effectiveness of the labels.

We first will conduct a pilot study via MTurk before fielding a larger study with TESS, another online panel provider.

We have now completed the MTurk pilot. The current revision is for the TESS online survey fielded through the NORC AmeriSpeak panel.

B. *Provide a sample size and a justification as to how you arrived at that number. If you use screening procedures to arrive at a final sample, distinguish the screening sample size from the enrolled sample size; a table may be helpful. For electronic survey studies involving online recruitment and survey completion: consider how you will set controls on how many people will join your study.*

The MTurk inititial samplew will be 400 people (100 in the control arm, 150 in the experimental arm 1, 150 in experimental arm 2. The TESS sample will be 5,000 people (divided evenly across the arms). The TESS study will include the online ordering task and the larger sample size reflects the variation and large standard deviations associated with online ordering tasks.

C. *Does your study meet the NIH definition of "clinical trial":* ""**A research study in which one or more human subjects are prospectively assigned to one or more interventions** (which may include placebo or other control) to evaluate the effects of those interventions on health-related biomedical or behavioral outcomes"? *If yes, the study must be listed on clinicaltrials.gov, study personnel must complete GCP training, and federally funded studies must post consent forms on approved sites, like clinicaltrials.gov.*

No.

IV. Participants:

Describe the study participants and the population from which they will be drawn. Specify the inclusion and exclusion criteria. If you plan to include children, note their ages and whether you will include children in foster care or who are wards of the State. Note if the participants are particularly vulnerable in terms of cognitive limitations, education, legal migration status, incarceration, poverty, or some combination of factors.

A. **Inclusion Criteria:**

18 years old or older, living in the United States.

B. **Exclusion Criteria:**

Less than 18 years old, not living in the United States.

NOTE*: If you are recruiting participants or receiving, accessing, or using data from a U.S. health care provider, HIPAA review is likely to be required. If you plan to bring identifiable health information from a foreign country to a U.S. covered entity (e.g., lab at the Hopkins SOM), HIPAA may be triggered. Check "yes" to the HIPAA question in the PHIRST application.*

V. Study Procedures:

In this section, provide details of your procedures, particularly as they relate to human subjects. If this is a multi-center study, make the role of JHSPH clear. If you will collaborate with other institutions or organizations, or plan to subcontract JHSPH responsibilities to others, make clear their responsibilities in the Study Oversight section of this document. Be aware that all recipients of federal funding for non-exempt human subjects research must have a Federal Wide Assurance (FWA) , which is a promise to comply with human subjects research regulations.

If the JHSPH will serve as ***data coordinating center****, indicate in the sections below which procedures JHSPH will not be performing. Additional information regarding data coordinating centers is requested in a later section.*

If your study will develop in phases, address each item below by phase.

A. **Recruitment Process:**

1. *Describe how you will identify, approach, and inform potential participants about your study. Include details about who will perform these activities and their qualifications*.

   1. The survey job will be posted on MTurk and MTurk 'workers' will be able to click on it and complete the survey.

   2. For the TESS study, TESS works with NORC's AmeriSpeak panel, a probability-based online survey platform. TESS will field the study working with NORC to qualified panel members.

2. *Address any privacy issues associated with recruitment. If recruitment itself may put potential participants at risk (if study topic is sensitive, or study population may be stigmatized), explain how you will minimize these risks*.

   There are no privacy concerns. We will have no interaction with participants, the surveys are anonymous, and we will not collect any identifying information.

B. **Consent Process**:

1. *Describe the following details about obtaining informed consent from study participants. If a screening process precedes study enrollment, also describe the consent for screening*.

   a. *Who will obtain informed consent, and their qualifications*:

We will seek consent at the start of the MTurk Survey. We provide a brief description and offer the participants a question to consent or not. Those who do not consent will have the survey ended at that point.

We will not seek consent for the TESS survey because NORC AmeriSpeak Panel members have already agreed to participate in surveys, and for the surveys that do not cover sensitive topics study-specific consent is not generally required.

b. *How, where, and when the consent discussion(s) will occur*:

N/A

c. *The process for determining whether a potential participant meets eligibility criteria. If you will collect personally identifiable information for screening purposes, collect only data needed for this purpose and explain what will happen to the data for individuals who are not eligible*:

MTurk: We ask participants their state/territory of residence and their age. Those who meet exclusion criteria will have their survey terminated. For the TESS study, the survey will not be sent to panel members who do not meet inclusion criteria.

d. *Whether you will obtain a signature from the participant or will use an oral consent process*:

No.

e. *Whether you will obtain a legally authorized representative's signature for adults lacking capacity*:

No.

f. *If children are included in the study, if and how you will obtain assent from them*:

N/A

g. If children are included in the study, how you will obtain permission for them to participate from their parent, legal guardian, or other legal authority (if child is in foster care or under government supervision). If any of the children are "wards of the state", additional regulatory requirements will apply:

N/A

h. *If you are seeking a waiver of informed consent or assent, the justification for this request*:

N/A

i. *Whether you will include a witness to the consent process and why*:

N/A

j. *If the language is unwritten, explain how you will communicate accurate information to potential participants and whether you will use props or audio materials*:

N/A

2. Identify the countries where the research will take place, and the languages that will be used for the consent process.

Country	Consent Document(s) (Adult Consent, Parental Permission, Youth Assent, etc.)	Languages
US	Consent **has already been granted by AmeriSpeak panel members**	English

C. **Study Implementation**:

1. *Describe the procedures that participants will undergo. If complex, insert a table below to help the reviewer navigate*.

   Participants will complete an approximately 5 minute annonymous online survey. Participants will be randomized to one of 3 conditions. In the first condition they will see 1 version of the menu with green 'low climate impact' labels. They will select an item to order then answer a serious of questions about the label. They will answer demographic questions, then the survey will end. In the second condition they will see 1 version of the menu with red 'high climate impact' labels. They will select an item to order then answer a serious of questions about the label. They will answer demographic questions, then the survey will end. In the third condition, the participants will see a 'control' menu with QR codes. They will then answer questions about the label. Next they will see both versions of the menu from prior arms (in random order) and answer questions about the label. Finally they will answer demographic questions and then the survey will end.

   In the TESS study, participants will be randomized to 1 of 3 arms, but the control arm will ONLY see the QR code menu and will not see the other labels. They also will not have to answer demographic questions as that information is already collected for NORC AmeriSPEAK participants.

2. *Describe the number and type of study visits and/or contacts between the study team and the participant, how long they will last, and where/how they will take place*.

   Particiapnts will take the survey, online on a computer or cell phone, at a place of their choosing. They will only take the survey 1 time. The survey is expected to last <5 minutes. There is no direct contact between the study team and the participant.

3. *Describe the expected duration of the study from the perspective of the individual participant and duration overall*.

   Approximately <5 minutes

4. *Provide a brief data analysis plan and a description of variables to be derived.*

The primary outcome measures are: (1) whether the respondent orders a low-GHGE menu item, and (2) whether the participant orders a meal that meets the nutrition criteria to be considered 'healthy'. Healthy items are defined as having a Nutrition Profile Index score of >64. The key independent variable will be a three-category indicator of the experimental arm to which the participant is randomized. Using logistic regressions, we will explore differences in primary and secondary outcomes between sustainability label conditions (arms 2 and 3) vs. the QR label control, and between the label conditions (arm 2 vs. arm 3). We will link publicly available nutrition information for the menu items to categorize items ordered as healthy vs. not healthy. We will compare the actual and perceived healthiness of the items ordered using Nutrition Profile Index scores. We will also explore potential effect modification based on sex, age, race, income, education, and climate change concern.

5. *Answer the following* ***if they are relevant to your study design****:*

   A. *If the study has different arms, explain the process for assigning participants (intervention/control, case/control), including the sequence and timing of the assignment.*

      Randomization within the survey software will randomize participants to different study arms.

   B. *If human biospecimens (blood, urine, saliva, etc.) will be collected, provide details about who will collect the specimen, the volume (ml) and frequency of collection, how the specimen will be used, stored, identified, and disposed of when the study is over. If specimens will be collected for use in future research (beyond this study), complete the "Biospecimen Repository" section below.*

   C. *If genetic/genomic analyses are planned, address whether the data will be contributed to a GWAS or other large dataset. Address returning unanticipated incidental genetic findings to study participants.*

   D. *If clinical or laboratory work will be performed at JHU/JHH, provide the JH Biosafety Registration Number.*

E. *If you will perform investigational or standard diagnostic laboratory tests using human samples or data, clarify whether the tests are validated and/or the lab is certified (for example is CLIA certified in the U.S.).* ***For clinical tests of human biospecimens, no results may be returned unless completed in a certified lab.*** *Explain the failure rate and under what circumstances you will repeat a test. For all human testing (biomedical, psychological, educational, etc.), clarify your plans for reporting test results to participants and/or to their families or clinicians. Address returning unanticipated incidental findings to study participants*.

F. If your study involves medical, pharmaceutical or other therapeutic intervention, provide the following information:

a. *Will the study staff be blind to participant intervention status*?

b. *Will participants receive standard care or have current therapy stopped*?

c. *Will you use a placebo or non-treatment group, and is that justifiable*?

d. *Explain when you may remove a participant from the study*.

e. What happens to participants on a study in which there is a medical intervention when the study ends? Will participants continue to have access to the study intervention? What happens if they leave the study early?

f. *Describe the process for referring participants to care outside the study, if needed*.

VI. Data Custody, Management, Security, and Confidentiality Protections: *Data security and management plans must meet institutional standards. If you need assistance, contact jhsph_cybersecurity@jhu.edu.*

Investigators are responsible for ensuring the security of data from the time of collection, through any transfers from one system to another, analysis, sharing, storage, and ultimate archiving and disposal. The questions below seek to elicit your plans for these protections. Feel free to add information.

1. **Data Sources**: Identify the source(s) of data.

☒ Participant/Parent-Guardian/Legally Authorized Representative
☐ JHM medical records (from Epic)

Note: Please complete the ***Data Trust Risk Tiers Calculator*** *available on the Applications and Forms page on the JHSPH IRB website: https://www.jhsph.edu/offices-and-services/institutional-review-board/applications-and-forms/, and upload a copy of the those documents to the "Miscellaneous" section of your PHIRST application. In addition, review the Data Protection Attestation for Research and/or Healthcare Operations at the link below and certify your attestation of compliance to those requirements: (*https://intranet.insidehopkinsmedicine.org/privacy_office/_docs/additional_information/Data%20Protection%20Attestation.pdf.

☐ **I certify my attestation of compliance to JHM Data Protection Requirements**

☐ non-JHM medical records
☐ Outside data provider (CMS, National Death Index, Insurance Co., etc.)
☐ Other existing records

2. **Data Content**: Will you collect, use, and/or record personal identifiers about study participants for any purpose? Please look at the list of identifiers in Question 3 to help answer this question. **Note: Limited Data Sets (including dates, ages, and zip codes) are considered to be "identifiable".**

☐ Yes Continue with Question 3
☒ No Skip to Question 9

3. Data Identification: Identify the Personally Identifiable Information (PII)/Protected Health Information (PHI) you will access/collect by checking the boxes below for "Recruitment" and "Study Data" needs:

Recruitment	Study Data	PII/PHI to be accessed/collected
☐	☐	Name, signature, initials or other identifiable code
☐	☐	Geographic identifier (address, GPS location, etc.)
☐	☐	Dates (birth, death, clinical service, discharge, etc.)
☐	☐	Contact information (phone number, email address, etc.)
☐	☐	Identification Numbers (SSN, driver's license, passport, etc.)
☐	☐	Health Records Identifiers (medical record #, insurance plan, etc.)
☐	☐	Text of clinical record notes
☐	☐	Device identifiers (implants, etc.)
☐	☐	Internet identifiers (IP address, social media accounts, etc.)
☐	☐	Biometric identifiers (fingerprints, retinal scan, voice print, etc.)
☐	☐	Audio Recordings
☐	☐	Video or full-face photographic images
☐	☐	Genomic / Genetic data
☐	☐	Other identifiers: (list here)

4. Identifiers: If you have checked any of the boxes above, how will you protect personal identifiers?

☐ Will delete all identifiers (explain **when** you will delete identifiers):

☐ Will separate identifiers from analytic data and will store the link/code. *Please explain where you will store link/code:*

☐ Will use a method to make it harder to connect the data with the study participant (jiggering date, use other methods to obfuscate, etc.). *Please explain:*

5. Will you obtain consent (or Authorization if governed by HIPAA) from participants for this study?

☐ Yes

☐ No

6. Data Transit Plans and Protections: Identifiable data may transfer, sometimes with multiple steps, from mechanisms for collection to storage. For example, participants may complete a web-based survey, which is then downloaded to a storage platform. Briefly identify these steps and the protections for each step (including encryption used at each step).

☐ Will delete all identifiers prior to transfer

☐ Will separate identifiers from analytic data and will store the link/code prior to transfer. *Please explain where you will store link/code:*

☐ Other:

7. Device(s) used for <u>data collection</u>: Identify the computing device(s) being used for identifiable data receipt/collection. Check all that apply.

☐ Provided or managed by JHSPH IT

☐ Study-provided, and not managed by JHSPH IT. These must include the following protective controls:

- Data encrypted while "at rest" (on a storage device)
- Security patches and updates are routinely or automatically applied
- Devices have access controls so that:
  - Each person accessing the device is uniquely identified (username)
  - Passwords are sufficiently strong to prevent compromise
  - All access is logged and recorded
  - Unauthorized access is prevented
- Approved access list is reviewed periodically for correctness
- ☐ Other. Specify:

8. Data Collection: Describe the format of data received/collected. Check all that apply.

☐ Paper/Hard Copy (must be secured in transit and placed in a secure cabinet/room)

☐ Audio recording

☐ Video recording

☐ Received directly by research team member and entered into file/database

☐ Mobile or Web App (custom developed). *Review <u>guidance</u> and provide attestation of compliance*

☐ Mobile or Web App (purchased). *Specify product and version*:

☒ Online survey. *Specify mechanism/platform*: Qualtrics/ MTurk **/ NORC AmeriSpeak panel**

☐ 3rd party collector. *Specify*:

☐ Existing data shared with JHSPH by data provider via electronic access/transfer

☐ Duplicate and backup copies will be secured with same rigor as original data

☐ Other. *Specify*:

9. Devices/Platforms used for Analysis, Storage, Processing: Identify where the identifiable or de-identified data will be analyzed/stored. Check all that apply.

☒ Pre-approved storage and analysis platforms managed by JH/JHSPH for which security and risk mitigation measures are known.

Identify preapproved storage platform(s) being used:

**JHM Preferred:**	**Other Approved**:	
☐ JH SAFE Desktop	☒ JH OneDrive/JHSPH OneDrive	☐ JHSPH HPCC
☐ JH PMAP	☐ JHSPH Shares	☐ JHM/JHSPH Qualtrics
	☐ JHSPH Sharepoint	☐ MARCC Secure Environment
	☐ JHU RedCap	☐ JH IT-managed Network Storage

____

☐ Platform(s) not managed by JH/JHSPH, not pre-approved, and require a risk assessment review from JHSPH Data Security. Describe:

Describe risk mitigation measures in place:

Note: The following are examples of platforms/storage solutions that are **not pre-approved to store identifiable information** and require a risk assessment from JHSPH Data Security.

- Other solutions not managed by IT@JH, e.g., commercial cloud storage (Box, Dropbox, iCloud, personal OneDrive, Google Drive, Amazon storage, etc.)
- JHU Independent Departmental Servers
- Local Computer owned by JH
- Other computers or devices owned/managed by study team members and used for other than secure web access
- USB/Portable data storage device

10. Access to Data and Access Controls: How will you ensure that only authorized individuals can access the data? What access controls will you put into place to ensure that only authorized individuals may access and use the data. (For example, OneDrive *guidance* illustrates how to share files with "people you specify". *JHSPH-Shares* addresses providing permissions to individual people.) Check all that apply. Note: If you need assistance implementing secure access controls, contact *jhsph_cybersecurity@jhu.edu.*

☒ Will provide access to data in accordance with OneDrive/JHSPH-Shares guidance posted on JHU IT websites

☐ Will use secure access controls to limit access to individual-level data

☐ Will use secure access controls to provide other researchers controlled access only to aggregated study data

11. Data Sharing: Clarify if data are to be shared externally with third parties, including sponsors and other investigators, and whether only aggregated data will be shared, or if you will share individual-level data. Describe sharing and protection plans for that sharing, including the proposed use of data agreements.

Consider the following:

- *Information about your data sharing in the consent forms*
- *Information about data sharing laws in the country where data will be collected, and if they limit sharing, how you will comply with those limitations?*
- *Whether data will be shared in aggregate only, or individual level data*
- *Whether you plan to make the data publicly available, and in what form.*

☐ Will not share data with outside investigators

☐ Will share aggregated data only

☒ Will share individual-level data without identifiers

☒ Will deposit data into an existing data repository for future research (e*xplain*):

☐ Other sharing information: **TESS funded studies require that data be made publicly available 1 year after the study is completed. All data will be de-identified and totally anonymous.**

12. Duration and Destruction: Explain how long data will be retained and the plan for eventual return, deidentification or destruction of data, including moving data to an archive.

Data will be retained for at least 5 years. All data is anonymous.

A. <u>Certificate of Confidentiality</u>:

All NIH studies include Certificate of Confidentiality (C of C) protections with the grant; the consent form must include the C of C language provided in our template. Other funders may obtain C of C protections through NIH. (https://grants.nih.gov/policy/humansubjects/coc.htm)

Does the study have Certificate of Confidentiality protections? Yes ☐ No ☒

VII. <u>Risks of the Study</u>:

A. *Describe the risks, discomforts, and inconveniences associated with the study and its procedures, including physical, psychological, emotional, social, legal, or economic risks, and the risk of a breach of confidentiality. Include risks beyond individuals to include the study population as a group and community risks. <u>Ensure that the risks described in the consent documents are consistent with the risks outlined in the research plan</u>.*

Breach of confidentiality is a potential risk **though given data security procedures at NORC and the fact that they will only share totally de-identified data with us, that risk is very very low**.

B. *Describe steps you will take to mitigate or minimize each of the risks described above. Include a description of your efforts to arrange for care or referral for participants who may need it.*

We are not collecting any identifying information and will not be collecting IP Address information either. We will have no way of identifying the participant or their location.

C. *Describe the anticipated frequency and severity of the harms associated with the risks identified above; for example, if you are performing "x" test/assessment, or dispensing "y" drug, how often do you expect an "anticipated" adverse reaction to occur in a study participant, and how severe do you expect that reaction to be?*

D. *Describe the research burden for participants, including time, inconvenience, invasion of privacy in the home, out of pocket costs, etc*.

The participants will participate in one approximately 5 minute online survey at a time convenient to them.

E. *Describe how participant privacy, and if relevant – family privacy - will be protected during data collection if sensitive questions are included in interviews, or if study visits occur in the home setting.*

N/A

VIII. Direct Personal and Social Benefits:

A. *Describe any potential direct benefits the study offers to participants ("payment" for participation is not a direct personal benefit).*

Participants may learn something new about the connection between food choices and climate change.

B. *Describe potential societal benefits likely to derive from the research, including value of knowledge learned.*

We may produce new evidence about the potential for sustainability menu labels to incentivize sustainable and healthy food choices. This would help address both diet quality and food sytems related climate change.

IX. Payment or Token of Appreciation:

A. *Do you plan to provide a non-monetary token of appreciation (food, soap, tea, chlorine tablets, etc.) to study participants? If no payment is provided, the JHSPH IRB strongly encourages providing such tokens. If yes, please describe below.*

Participants are compensated by the respective survey firms according to their guidelines.

B. *If you plan to provide a monetary payment, describe the form, amount, and schedule of payment to participants. Reimbursement for travel or other expenses is not "payment," and if the study will reimburse, explain*.

Level of compensation is set by the survey firm according to their guidelines.

C. *Include the possible total remuneration and any consequences for not completing all phases of the research.*

None

X. Study Management:

A. **Oversight Plan:**

1. *Describe how the study will be implemented. List all parties, including collaborators and subcontractors, who will be "engaged" in the human subjects research project and their roles .*

    Dr. Julia Wolfson, PhD MPP is the PI of the study and will lead data collection and analysis

We will provide a link to the programmed survey (in Qualtrics) to MTurk and respondents will be able to access the survey. For the TESS survey, we provide the questions to TESS and they program the survey into other surveys they are fielding via the NORC Amerispeak panel.

Other collaborators on the research team who will be engaged in interpretation of study results and writing up results for publication, but will not be engaged in data collection of analysis are:

Dr. Cindy Leung, ScD. University of Michigan SPH

Dr. Jennifer Falbe, ScD, University of California Davis

Dr. Ashley Gearhardt, PhD, University of Michigan

Dr. Aviva Musicus, PhD, Harvard University SPH

2. *What are the qualifications of study personnel implementing the project*?

    PhD in Public Health and 7 years experience conducting online survey research

3. *How will non-professional personnel (data collectors) involved with the data collection and analysis be trained in human subjects research ethical protections? (Use the JHSPH Ethics Field Training Guide available on the JHSPH IRB website. If the study is a clinical trial, consider using the JHSPH Good Clinical Practice (GCP) For Social and Behavioral Research Field Guide).*

4. *If the JHSPH PI is responsible for data collection and will not personally be on-site throughout the data collection process, provide details about PI site visits, the supervision over consent and data collection, and the communication plan between the PI and study team*.

B. **Protocol Compliance and Recordkeeping:**

Describe how you plan to ensure that the study team follows the protocol and properly records and stores study data collection forms, IRB regulatory correspondence, and other study documentation. (For assistance, contact: housecalls@jhu.edu *Please provide information about study oversight to ensure compliance with IRB approval and regulatory and institutional requirements. If the study team does not follow study procedure, what is your plan for reporting protocol non-compliance?*

Data and IRB correspondence and other study documents will be stored on password protected computers.

C. **Safety Monitoring:**

1. *Describe how participant safety will be monitored as the study progresses, by whom, and how often. Will there be a medical monitor on site? If yes, who will serve in that role and what is that person's specific charge?*

2. *If a Data Safety Monitoring Board (DSMB), or equivalent will be established, describe the following*:

    a. *The DSMB membership, affiliation and expertise*.

    b. *The charge or charter to the DSMB*.

    c. *Plans for providing DSMB reports to the IRB*.

3. *Describe plans for interim analysis and stopping rules, if any*.

D. **Reporting Unanticipated Problems/Adverse Events (AEs) to the IRB (*all studies must complete this section*)**:

NOTE: The IRB does not require PROMPT reporting of all AEs, only those that are ***unanticipated, pose risk of harm to participants or others, and are related to the study****.*
Anticipated *AEs may be reported with the Continuing Review/Progress Report.*

Describe your plan for reporting to the JHSPH IRB, local IRBs, and (if applicable) to the sponsor. Include your plan for government-mandated reporting of child abuse or illegal activity.

While we do not expect any adverse events, should any occur we will report them immediately to the IRB.

E. **Other IRBs/Ethics Review Boards:**

If other IRBs will review the research, provide the name of each IRB/ethics review board and its Federal Wide Assurance number, if it has one (available on OHRP's website at http://www.hhs.gov/ohrp/assurances). ***For federally funded studies, subrecipients MUST have a Federal Wide Assurance (FWA) number from the OHRP. The IRB overseeing the subrecipient should be registered with the OHRP. The JHSPH IRB will not have oversight responsibility for international subrecipients, and generally will not oversee data collection at external U.S. institutions Please contact jhsph.irboffice@jhu.edu with questions.***

Non-JHSPH IRB/REC	FWA Number

F. **"Engaged" in Human Subjects Research:**

For studies that involve collaboration with non-JHSPH institutions, complete the chart below by describing the collaboration and the roles and responsibilities of each partner, including the JHSPH investigator. This information helps us determine what IRB oversight is required for each party. Complete the chart for all multi-collaborator studies.

Insert collaborator names and FWA numbers, if available. Note who will be "engaged" in human subjects research by filling in the following table:

	JHSPH		
For federally funded studies, collaborators' FWA	00000287		
Primary Grant/Contract Recipient			
Grant/Contract Subrecipient			
Hiring Data Collectors			
Training Data Collectors			
Obtaining Informed Consent and/or Identifiable Data			
Accessing/Analyzing Identifiable Data			
Overseeing storage, access and use of biospecimens			

COMPLETE THE FOLLOWING SECTIONS WHEN RELEVANT TO YOUR STUDY:

XI. Secondary Data Analysis of Existing Data:

A. **Study Design**:

1. *Describe your study design and methods. The study design must relate to your stated aims/objectives.*

2. *Provide an estimated sample size and an explanation for that number*.

3. *Provide a brief data analysis plan and a description of variables to be derived.*

B. **Participants:**

1. *Describe the subjects who provided the original data and the population from which they were drawn.*

Note: If you are receiving, accessing, or using data from a U.S. health care provider, the need for HIPAA review is likely. If you plan to bring identifiable health information from a foreign country to a U.S. covered entity (e.g., lab at the Hopkins SOM), HIPAA may be triggered. If either of these conditions is met, check "yes" to the HIPAA question in the PHIRST application.

2. *If you plan to analyze human specimens or genetic/genomic data, provide details about the source of those specimens and whether they were collected using an informed consent document. If yes, explain whether your proposed use is "consistent with" the scope of the original consent, if it potentially introduces new analyses beyond the scope of the original consent, and/or if it introduces new sensitive topics (HIV/STDs, mental health, addiction) or cultural/community issues that may be controversial.*

3. *Explain whether (and how) you plan to return results to the participants either individually or as a group*.

XII. <u>Oversight Plan for Student-Initiated Studies</u>:

A. *For student-initiated studies, explain how the PI will monitor the student's adherence to the IRB-approved research plan, such as communication frequency and form, training, reporting requirements, and anticipated time frame for the research. Describe who will have direct oversight of the student for international studies if the PI will not personally be located at the study site, and their qualifications.*

B. *What is the data custody plan for student-initiated research? (Note: Students may not take identifiable information with them when they leave the institution.)*

XIII. <u>Creation of a Biospecimen Repository</u>:

Explain the source of the biospecimens, if not described above, and what kinds of specimens will be retained over time. Clarify whether the specimens will be obtained specifically for repository purposes, or will be obtained as part of the core study and then retained in a repository.

A. Describe where the biospecimens will be stored and who will be responsible for them.

B. Describe how long the biospecimens will be stored, and what will happen at the end of that period.

C. *Explain whether the biospecimens will be shared with other investigators, inside and outside of JHU, how the decision to share will be made, and by whom. Include your plans, if any, for commercial use. Also explain how downstream use of the specimen will be managed, and what will happen to left-over specimens*.

D. Describe whether future research using the biospecimens will include specimen derivation and processing (cell lines, DNA/RNA, etc.), genomic analyses, or any other work which could increase risk to participants. Explain what additional protections will be provided to participants.

E. *If future research could yield unanticipated incidental findings (e.g., an unexpected finding with potential health importance that is not one of the aims of the study) for a participant, do you intend to disclose those findings to the study participant? Please explain your position.*

F. *Explain whether the specimens will be identifiable, and if so, how they will be coded, who will have access to the code, and whether the biospecimens will be shared in linked (identifiable) form*.

G. *Explain whether the repository will have Certificate of Confidentiality protections.*

H. *Explain whether a participant will be able to withdraw consent to use a biospecimen, and how the repository will handle a consent withdrawal request.*

I. *Describe data and/or specimen use agreements that will be required of users. Provide a copy of any usage agreement that you plan to execute with investigators who obtain biospecimens from you.*

XIV. <u>Data Coordinating Center</u>:

Complete if JHSPH serves as the Data Coordinating Center.

A. *How will the study procedures be developed?*

B. *How will the study documents that require IRB approval at each local site be developed? Will there be some sort of steering or equivalent committee that will provide central review and approval of study documents, or will template consent forms, recruitment materials, data collection forms, etc. be developed by and provided to the local sites by the coordinating center without external review?*

C. *Will each local clinical site be overseen by its own IRB with an FWA, or will a Single IRB review the study? State whether the coordinating center will collect IRB approvals and renewals from the clinical centers; if not, explain why.*

D. *How will the coordinating center provide each local site with the most recent version of the protocol and other study documents? What will be the process for requesting that these updates be approved by local clinical center IRBs?*

E. *What is the plan for collecting data, managing the data, and protecting the data at the coordinating center?*

F. *What is the process for reporting and evaluating protocol events and deviations from the local sites? Who has overall responsibility for overseeing subject safety: the investigators at the recruitment site, the Coordinating Center, the Steering Committee, or a Data and Safety Monitoring Board (DSMB)? Is there a DSMB that will evaluate these reports and provide summaries of safety information to all the reviewing IRBs, including the coordinating center IRB? Please note that if there is a DSMB for the overall study, then the coordinating center PI does not have to report to the coordinating center IRB each individual adverse event/problem event that is submitted by the local site PIs.*

G. *Some FDA regulated studies have different AE reporting criteria than that required by the IRB (IRB Policy No. 103.06). How will you reconcile the different requirements, and who is responsible for this reconciliation?*

H. *Who is responsible for compliance with the study protocol and procedures and how will the compliance of the local sites be monitored and reviewed? How will issues with compliance be remedied?*

XV. <u>Drug Products, Vitamins, Food and Dietary Supplements:</u>

Complete this section if your study involves a drug, botanical, food, dietary supplement or other product that will be applied, inhaled, ingested or otherwise absorbed by the study participants. If you will be administering drugs, please upload the product information.

A. *List the name(s) of the study product(s), and the manufacturer/source of each product.*

**Name of Study Product**	**Manufacturer/Source**

B. *List each study product by name and indicate its approved/not approved status.*

**Approved by the FDA and Commercially Available**	**Approved by Another Gov’t Entity (provide name)**	**Cleared for Use at Local Study Site**

C. *If your study product has an Investigational New Drug (IND) application through the U.S. Food and*

Drug Administration, provide the IND number, and the Investigators Brochure.

a. *Who will hold the IND?*

D. *If your study product is a marketed drug, provide the package inserts or other product information. If the study product WILL NOT be used for its approved indication, dose, population, and route of administration, provide a detailed rationale justifying the off-label use of the study product.*

E. *If the study product does not require FDA approval (e.g., dietary supplements, botanicals, products not subject to the U.S. FDA, etc.), provide safety information (as applicable) and a certificate of analysis.*

F. *Explain who will be responsible for drug management and supply, labeling, dispensing, documentation and recordkeeping. Complete and upload into PHIRST the Drug Data Sheet available on the JHSPH IRB website at www.jhsph.edu/irb.*

G. *What drug monitoring and/or regulatory oversight will be provided as part of the study? Please describe.*

XVI. Medical Devices:

*Complete this section if your study will involve an approved or investigational medical device (**diagnostic**, non-significant risk, significant risk).*

A. *List the name(s) of the study product(s), the manufacturer/source of each product, and whether or not it is powered (electric, battery). Provide product information. If it is electric, upload documentation of clinical engineering approval or its equivalent from a local authority, to ensure that the device is in good working order.*

Name of Study Product	Manufacturer/Source	Powered?

B. *List each study product by name and indicate its status as approved by a government authority or not approved.*

**Approved by the FDA and Commercially Available**	**Approved by Another Gov't Entity (provide name and approval information)**	**Not Approved**

C. *If your investigational device is Exempt from the FDA IDE regulations, explain which section of the code applies to your device and why it meets the criteria provided. If it is a* ***diagnostic device****, provide pre-clinical information about the sensitivity and specificity of the test and the anticipated failure rate. If you plan to provide the results to participants or their physicians, justify doing so, and explain how those results will validated (or not) against the current "gold standard".*

D. *If you believe the investigational device is not IDE exempt under 21CFR 812.2(c), but is a "Non-Significant Risk" device considered to have an approved IDE application, provide information from the manufacturer supporting that position.*

E. *If you are using an investigational device that is a Significant Risk Device, provide the IDE number given by the FDA, or if not under FDA jurisdiction, explain why it is appropriate to use this device in this study. Provide a description of the device, and upload a picture or manufacturing schematics into PHIRST. Provide any other information relevant to a determination of its safety to be used for the purposes outlined in this research plan.*
