# Supplementary material for: Effect of Climate Change Impact Menu Labels on Fast Food Ordering Choices Among US Adults: A Randomized Clinical Trial
Source: JAMA Netw Open. 2022 Dec 27;5(12):e2248320. doi: 10.1001/jamanetworkopen.2022.48320 (PMC9857560; doi:10.1001/jamanetworkopen.2022.48320)
Supplement: Supplement 2. — eFigure 1. Proportion of Respondents Who Reported Noticing Labels on Menu by Treatment Condition eFigure 2. Perceptions That Label Discouraged Consumption of Menu Items With High Impact on Climate Change eFigure 3. Predicted Probability of Selecting Sustainable Menu Item by Treatment Condition and Sex eFigure 4. Predicted Probability of Selecting Beef Hamburger, Plant-Based Hamburger, Chicken or Fish Item, or Salad by Experimental Condition eTable. Nutrition Profile Index Score and Calorie Content of Menu Items eFigure 5. Predicted Mean Nutrition Profile Index Score of Ordered Item by Experimental Condition [file jamanetwopen-e2248320-s002.pdf]

Supplemental Online Content

Wolfson JA, Musicus AA, Leung CW, Gearhardt AN, Falbe J. Effect of climate change impact menu labels on fast food ordering choices among US adults: a randomized clinical trial. *JAMA Netw Open*. 2022;5(12):e2248320. doi:10.1001/jamanetworkopen.2022.48320

eFigure 1. Proportion of Respondents Who Reported Noticing Labels on Menu by Treatment Condition
eFigure 2. Perceptions That Label Discouraged Consumption of Menu Items With High Impact on Climate Change
eFigure 3. Predicted Probability of Selecting Sustainable Menu Item by Treatment Condition and Sex
eFigure 4. Predicted Probability of Selecting Beef Hamburger, Plant-Based Hamburger, Chicken or Fish Item, or Salad by Experimental Condition
eTable. Nutrition Profile Index Score and Calorie Content of Menu Items
eFigure 5. Predicted Mean Nutrition Profile Index Score of Ordered Item by Experimental Condition

This supplemental material has been provided by the authors to give readers additional information about their work.

© 2022 Wolfson JA et al. *JAMA Network Open.*

eFigure 1. Proportion of Respondents Who Reported Noticing Labels on Menu by Treatment Condition

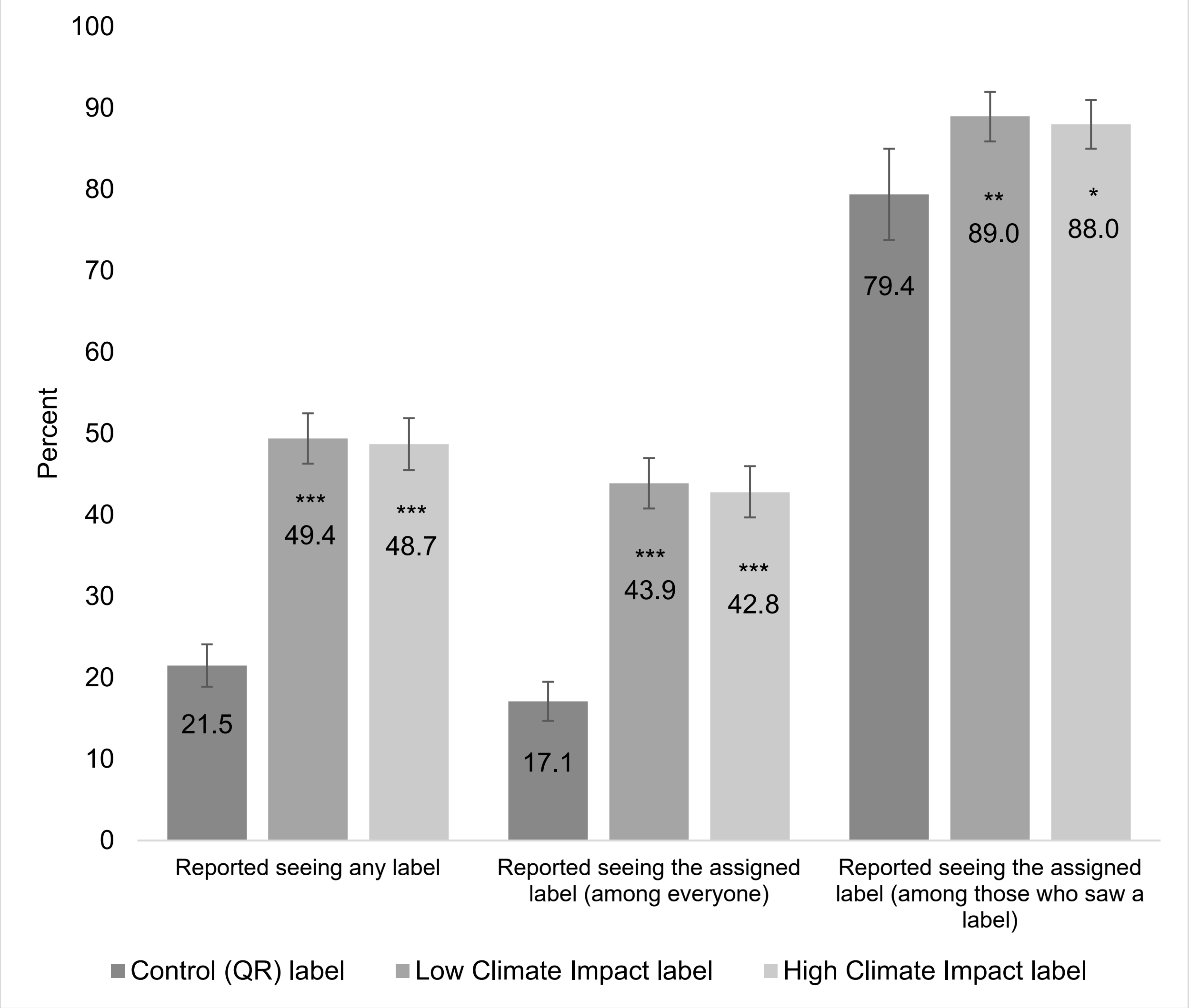

Note: Post estimation margins after weighted simple logistic regression. Difference from control label significant at * p<0.05; ** p<0.01 *** p<0.001. There were no significant differences between low- and high-climate-impact labels.

© 2022 Wolfson JA et al. *JAMA Network Open.*

eFigure 2. Perceptions That Label Discouraged Consumption of Menu Items With High Impact on Climate Change

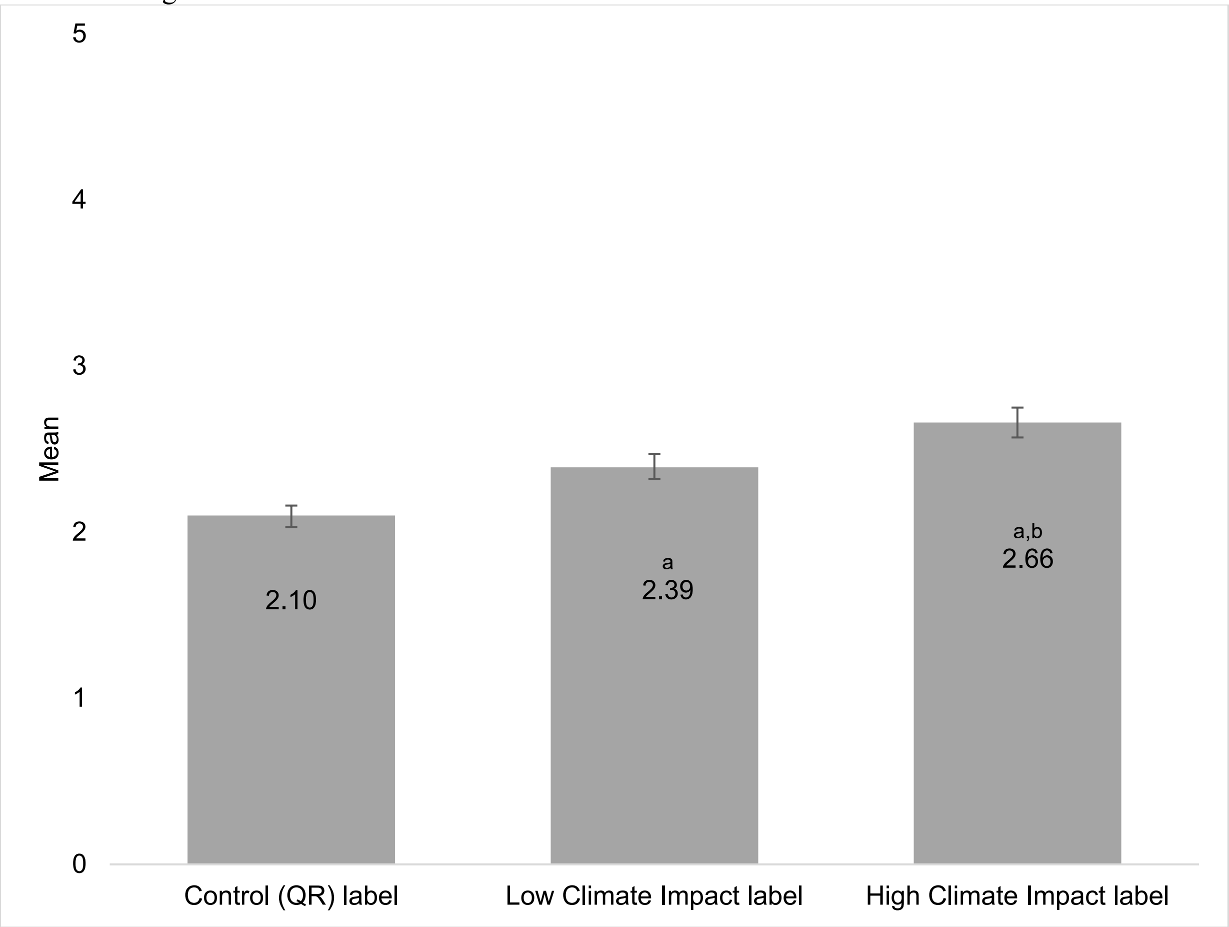

Note: Predicted marginal mean after simple linear regression. Y-axis is the mean value of responses on a 5-point scale from 1=strongly disagree to 5=strongly agree.

[a] Difference from control label significant at p<0.001

[b] Difference from low-climate-impact label significant at p<0.001

© 2022 Wolfson JA et al. *JAMA Network Open.*

eFigure 3. Predicted Probability of Selecting Sustainable Menu Item by Treatment Condition and Sex

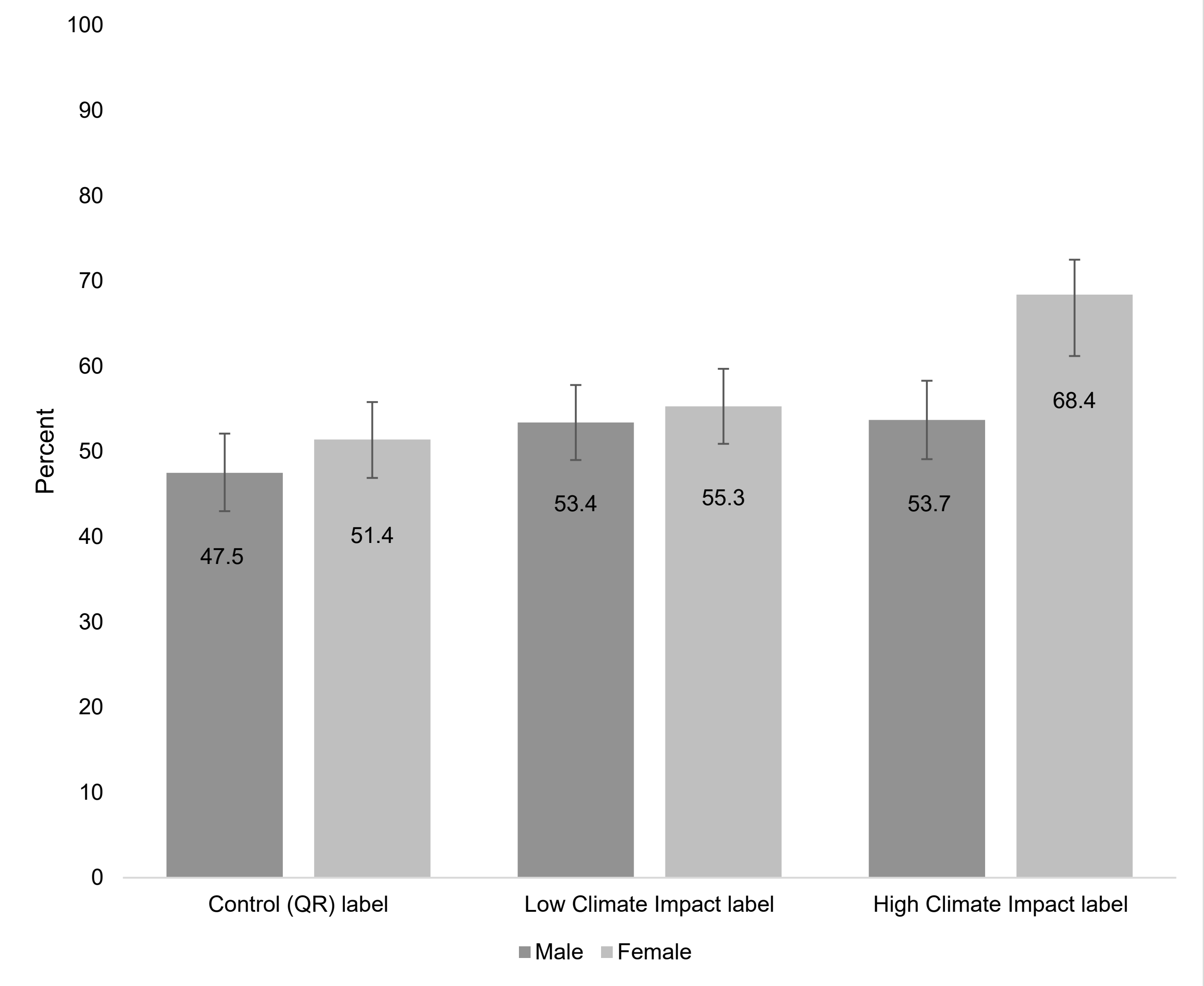

Note: Post estimation margins after weighted simple logistic regression with an interaction between experimental condition and gender (interaction p=0.023).

© 2022 Wolfson JA et al. *JAMA Network Open.*

eFigure 4. Predicted Probability of Selecting Beef Hamburger, Plant-Based Hamurger, Chicken or Fish Item, or Salad by Experimental Condition

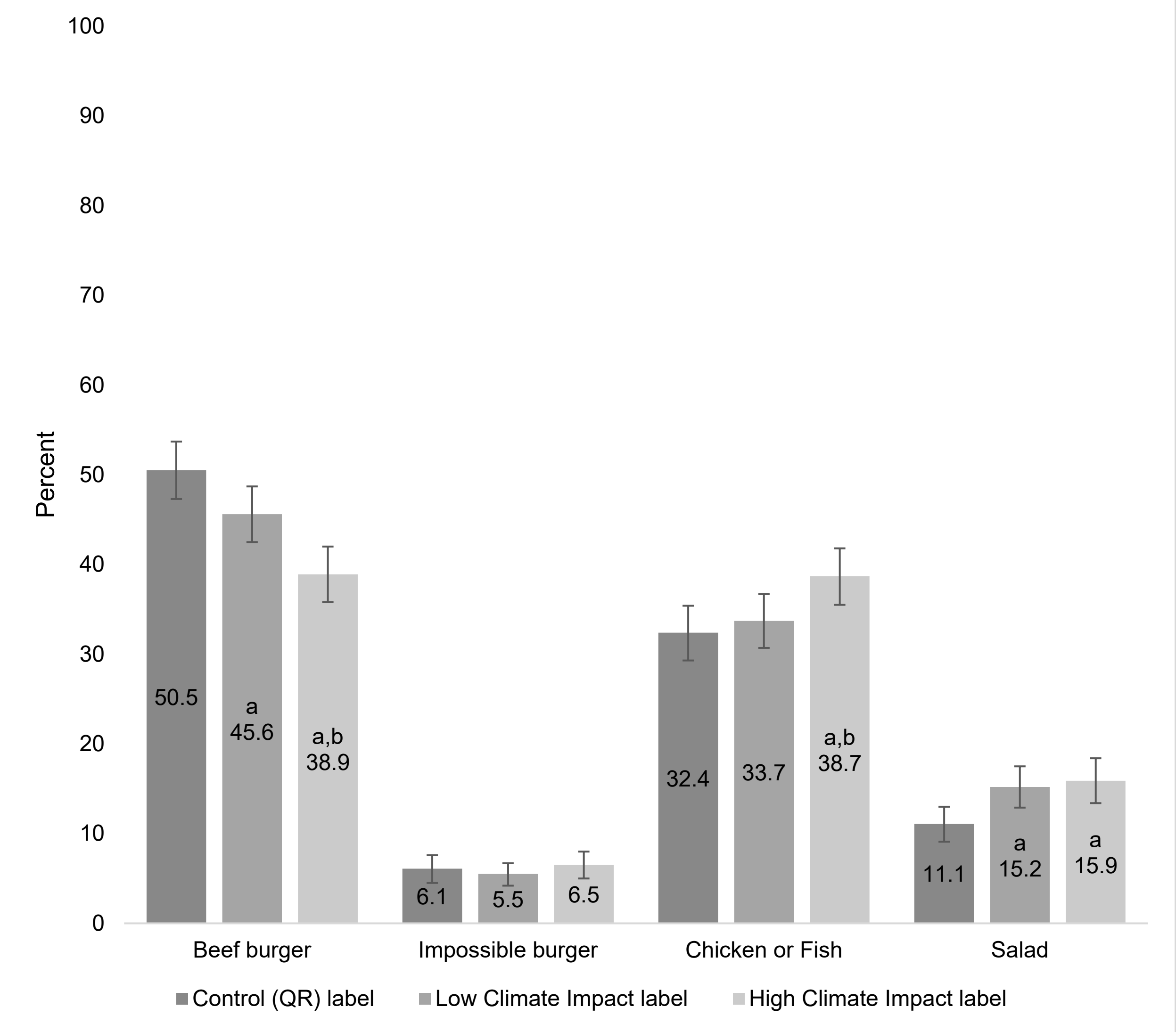

Note: Predicted probability from post estimation margins after weighted multinomial logistic regression.
[a] Difference from control condition (within food category) significant at p<0.05.
[b] Difference from low-climate-impact label condition (within food category) significant at p<0.05.

© 2022 Wolfson JA et al. *JAMA Network Open.*

eTable. Nutrition Profile Index Score and Calorie Content of Menu Items

Item	NPI Score	Calories[a]	Healthy (NPI score of ≥64)	Sustainable
Flame Grilled Burgers				
Whopper	58	670	No	No
Impossible™ Whopper	62	627	No	Yes
Whopper Jr.	58	340	No	No
Double Whopper with Cheese	54	1002	No	No
Bacon Double Cheeseburger	36	533	No	No
Cheeseburger	50	302	No	No
Chicken & Fish				
Crispy Chicken Sandwich	54	916	No	Yes
Spicy Crispy Chicken Sandwich	48	1076	No	Yes
Original Chicken Sandwich	58	684	No	Yes
Chicken Jr.	60	445	No	Yes
10 pc. Nuggets	54	482	No	Yes
Big Fish	56	561	No	Yes
Salads				
Chicken Garden Salad	56	870	No	Yes
Side Garden Salad	58	322	No	Yes

Note: Calorie and nutrient information taken from Burger King website (accessed on June 8, 2022) and used to compute NPI score. Serving size information was not available on the Burger King website and was taken from a PDF posted separately: https://company.bk.com/pdfs/nutrition.pdf

[a] Calorie information differs somewhat from calories displayed on the menus used in the online experiment. Calorie information for those menus were taken from the Burger King menu in November 2021, whereas nutrient and calorie information for analyses were gathered in June 2022.

© 2022 Wolfson JA et al. *JAMA Network Open.*

eFigure 5. Predicted Mean Nutrition Profile Index Score of Ordered Item by Experimental Condition

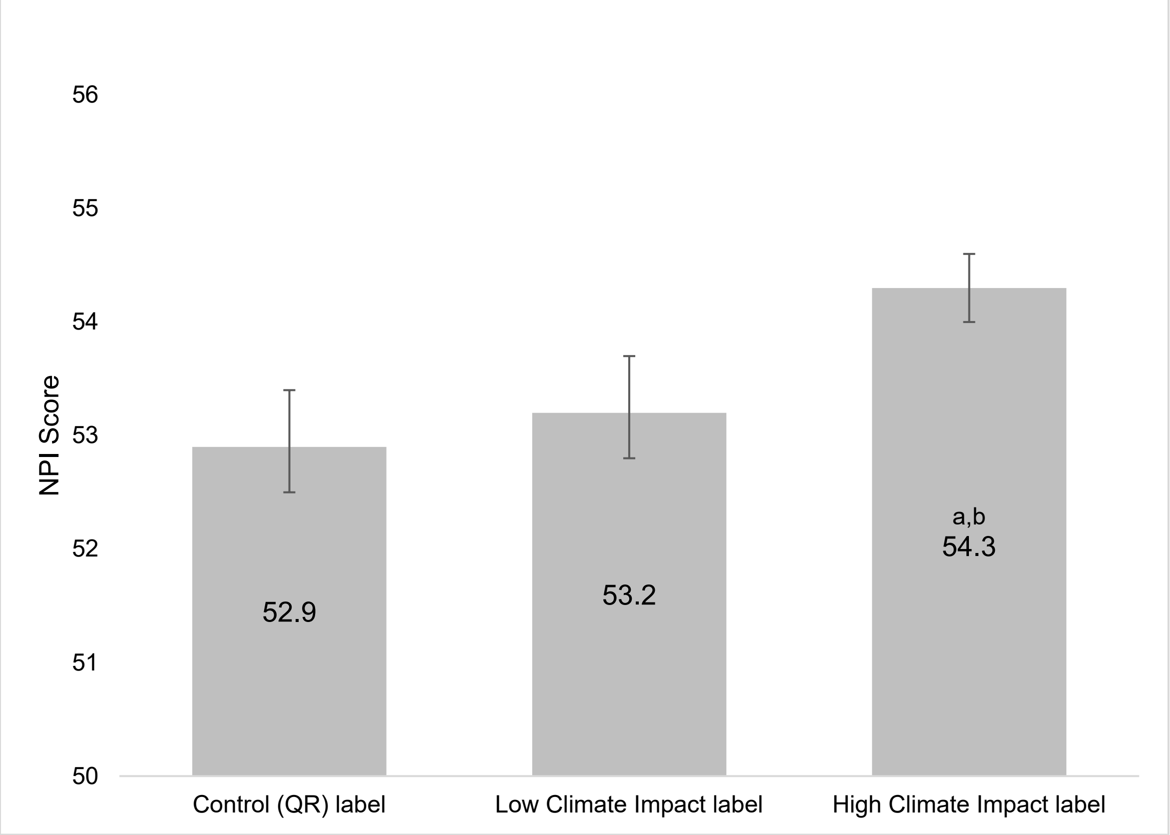

Note: Post estimation margins after weighted simple linear regression.
[a] Difference from the control label condition significant at p<0.001.
[b] Difference from the low-climate-impact label condition significant at p<0.001.

© 2022 Wolfson JA et al. *JAMA Network Open.*
