# Supplementary material for: Effect of Climate Change Impact Menu Labels on Fast Food Ordering Choices Among US Adults: A Randomized Clinical Trial
Source: JAMA Netw Open. 2022 Dec 27;5(12):e2248320. doi: 10.1001/jamanetworkopen.2022.48320 (PMC9857560; doi:10.1001/jamanetworkopen.2022.48320)
Supplement: Supplement 3. — Data Sharing Statement [file jamanetwopen-e2248320-s003.pdf]

Data Sharing Statement

Wolfson JA, Musicus AA, Leung CW, Gearhardt AN, Falbe J. Effect of climate change impact menu labels on fast food ordering choices among US adults: a randomized clinical trial. *JAMA Netw Open*. 2022;5(12):e2248320. doi:10.1001/jamanetworkopen.2022.48320

Data
Data available: Yes
Data types: Deidentified participant data
How to access data: After April 2023, data and study materials will be publicly available on the website for TESS (https://www.tessexperiments.org/paststudies)
When available: beginning date: 05-01-2023

Supporting Documents
Document types: Other (please specify)
Additional Information: Survey instrument and codebook
How to access documents: After April 2023, data and study materials will be publicly available on the website for TESS (https://www.tessexperiments.org/paststudies)
When available: beginning date: 05-01-2023

Additional Information
Who can access the data: Data and study materials will be publicly available for download. **Types of analyses:** Data will be available for public use without pre-approval needed. **Mechanisms of data availability:** Data will be publicly available at https://www.tessexperiments.org/paststudies .

© 2022 Wolfson JA et al. *JAMA Network Open*.
